# Association of general anesthesia exposure with risk of postoperative delirium in patients receiving transcatheter aortic valve replacement: a meta-analysis and systematic review

**DOI:** 10.1038/s41598-023-43548-2

**Published:** 2023-09-27

**Authors:** Ching-Chung Ko, Kuo-Chuan Hung, Yang-Pei Chang, Chien-Cheng Liu, Wan-Jung Cheng, Jheng-Yan Wu, Yu-Yu Li, Tso-Chou Lin, Cheuk-Kwan Sun

**Affiliations:** 1 Department of Medical Imaging, Chi Mei Medical Center, Tainan City, Taiwan; 2 Department of Health and Nutrition, Chia Nan University of Pharmacy and Science, Tainan City, Taiwan; 3 Department of Anesthesiology, Chi Mei Medical Center, Tainan City, Taiwan; 4 School of Medicine, College of Medicine, National Sun Yat-Sen University, Kaohsiung City, Taiwan; 5 Department of Neurology, Kaohsiung Municipal Ta-Tung Hospital, Kaohsiung Medical University, Kaohsiung City, Taiwan; 6 Department of Neurology, Kaohsiung Medical University Hospital, Kaohsiung Medical University, Kaohsiung City, Taiwan; 7 Department of Anesthesiology, E-Da Hospital, I-Shou University, Kaohsiung City, Taiwan; 8 Department of Nutrition, Chi Mei Medical Center, Tainan City, Taiwan; 9 Department of Anesthesiology, Chi Mei Medical Center, Chiali, Tainan City, Taiwan; 10 Department of Anesthesiology, Tri-Service General Hospital, National Defense Medical Center, Taipei City, Taiwan; 11 Department of Emergency Medicine, E-Da Dachang Hospital, I-Shou University, No. 305, Dachang 1St Road, Sanmin District, Kaohsiung City, Taiwan; 12 School of Medicine for International Students, College of Medicine, I-Shou University, Kaohsiung City, Taiwan

**Keywords:** Cardiology, Health care, Risk factors

## Abstract

The aim of this meta-analysis was to assess the association of general anesthesia (GA) exposure with the risk of POD in this patient population. Databases including Medline, EMBASE, Cochrane library, and Google Scholar were searched from inception to December 2022. Analysis of 17 studies published between 2015 and 2021 involving 10,678 individuals revealed an association of GA exposure with an elevated risk of POD [odd ratio (OR) = 1.846, 95% CI 1.329 to 2.563, *p* = 0.0003, I^2^ = 68.4%, 10,678 patients]. Subgroup analysis of the diagnostic methods also demonstrated a positive correlation between GA exposure and POD risk when validated methods were used for POD diagnosis (OR = 2.199, 95% CI 1.46 to 3.31, *p* = 0.0002). Meta-regression analyses showed no significant impact of age, male proportion, and sample size on the correlation between GA and the risk of POD. The reported overall incidence of POD from the included studies regardless of the type of anesthesia was between 0.8 and 27%. Our meta-analysis showed a pooled incidence of 10.3% (95% CI 7% to 15%). This meta-analysis suggested an association of general anesthesia with an elevated risk of postoperative delirium, implying the necessity of implementing appropriate prophylactic strategies against this complication when general anesthesia was used in this clinical setting.

## Introduction

Transcatheter aortic valve replacement (TAVR), which is a minimally invasive therapeutic procedure for aortic stenosis^1^, is typically performed in patients who are considered high-risk for traditional open-heart surgery^1^. TAVR has become increasingly popular in recent years due to its minimally invasive nature and high success rate^2,3^. Although it is conventionally conducted under general anesthesia (GA) with most patients being discharged from the hospital within a few days after the procedure^4^, postoperative delirium (POD) characterized by a fluctuation in mental status may occur^5,6^. POD occurs most commonly between postoperative days 1 and 3 when patients may present with confusion, a reduced awareness of the environment, disorientation, a disturbance of attention, and changes in behavior^7–9^. Following TAVR, the pooled incidence of POD was reportedly up to 8.1–9.8% in previous meta-analyses^10,11^. Not only may POD significantly prolong hospitalization and increase medical expenditure^12–15^, but it has also been shown to be an independent risk factor for mortality in patients undergoing TAVR^14–17^. Therefore, prevention of POD through pre-procedural identification of its risk factors may help in timely implementation of appropriate prophylactic and management strategies to improve the quality of patient care^12^.

The reported risk factors for POD following TAVR include advanced age, non-transfemoral (i.e., transapical/transaortic) access, the presence of carotid artery disease, male gender, stroke, current smoking, and history of atrial fibrillation^14,15^. Besides, GA exposure has been shown to be a potential risk factor for POD in this patient population^11^. Although avoidance of GA exposure is believed to prevent POD, findings from current literature remain inconclusive^18–23^. Two recent randomized controlled trials (RCTs) demonstrated no significant difference in the occurrence of POD between patients undergoing regional anesthesia and those receiving GA for orthopedic surgeries^24,25^. For patients undergoing TAVR, several retrospective studies reported the association of GA exposure with an elevated risk of POD^14,15,26,27^, while evidence from a RCT involving 438 patients indicated no increased risk of POD with the use of GA compared to local anesthesia/conscious sedation^28^.

Although a recent updated meta-analysis attempted to explore the relationship between GA exposure and POD risk^11^, inclusion of a limited number of patients (e.g., unadjusted data from 3555 patients and adjusted data from 1537 patients) may bias their results. Through focusing on general anesthesia and adopting a more comprehensive analytical approach, the current meta-analysis aimed at elucidating the association of GA exposure with the risk of POD in those undergoing TAVR.

## Methods

The protocol of the present meta-analysis was registered in PROSPERO (CRD42023398788). The presentation of the current study followed the Preferred Reporting Items for Systematic Reviews and Meta-Analyses (PRISMA) statement.

### Database search strategy

We searched four databases including Medline, Google Scholar, Embase, and Cochrane library for relevant studies from inception to February 10, 2023. The key words used for screening included: (“transcatheter aortic valve replacement” or “transcatheter aortic valve implantation” or “TAVR” or “TAVI” or “Aortic valve stenosis”) and (“general anesthesia” or “anesthesia” or “endotracheal intubation” or “inhalation agents”) and (“delirium” or “postoperative delirium” or “Confusion Assessment Method” or “acute brain failure” or “cognitive decline” or “altered mental status” or “cognitive dysfunction” or “organic brain syndrome” or “cognition impairment” or ”acute brain dysfunction”). There was no restriction on publication year or language when searching the databases. Reference lists of the retrieved studies were examined to identify potentially eligible articles that were not included on initial literature search. Supplementary Table 1 summarized the details on search strategies used in one of the databases (i.e., Medline).

### Studies selection and inclusion criteria

Two authors independently screened the titles and abstracts of the acquired articles based on predefined criteria before reading the full texts to make the final decision. Any disagreement on studies selection was resolved through discussion. Eligible articles must meet the following criteria: (1) Population: adults undergoing TAVR regardless of its site of access; (2) Intervention: GA was used for TAVR without restriction on the strategy of airway management (e.g., supraglottic airway devices or endotracheal intubation) or the anesthetics used (e.g., inhalation agents or intravenous anesthetics); (3) Comparison: local anesthesia or/and conscious sedation/monitored anesthesia care; (4) Outcomes: risk of POD, and (5) Type of article: RCTs or retrospective cohort studies considered eligible for the current meta-analysis.

Studies were excluded if they (1) recruited surgical or pediatric population; (2) did not report relevant details for risk calculation (e.g., events and total number); and (3) were published as letters, reviews, case reports, or conference abstracts.

### Data extraction, quality assessment, and certainty of evidence

Two independent authors extracted relevant details from the eligible articles, including the patient characteristics (i.e., age, male proportion, and body mass index), first author, year of publication, sample size, methods for diagnosing POD, incidence of delirium, ejection fraction, and country. A third author was involved for any disagreement on data extraction. When there was an overlap of patient populations between different studies, the study with a complete data set and/or with the largest estimated population was selected. If necessary, we emailed the authors of the included studies for missing information. The quality of individual studies was appraised with Newcastle–Ottawa Scale (NOS) for observational studies or the Cochrane Collaboration’s risk of bias tool (ROB 2.0) for RCTs. When using NOS, cohort studies being assigned more than seven points were considered low risk of bias. The overall certainty of evidence was assessed with the Grading of Recommendations Assessment, Development and Evaluation (GRADE) framework.

### Outcomes and subgroup analysis

The primary outcome of this study was the correlation of GA exposure with the risk of POD, while the pooled incidence of POD among patients receiving TAVR was the secondary outcome. Subgroup analysis was performed based on the methods for diagnosis of POD. Validated methods referred to the use of standardized tools designed specifically for diagnosing delirium, such as the Confusion Assessment Method (CAM), Confusion Assessment Method for the Intensive Care Unit (CAM-ICU), Nursing Delirium Screening Scale (NU-DESC), and criteria from the Diagnostic and Statistical Manual of Mental Disorders (DSM). Non-validated methods referred to diagnosis of POD based on non-standardized approaches, such as symptom evaluation or use of a chart-based delirium identification instrument.

### Statistical analysis

The primary outcome was presented as odds ratios (ORs) with 95% confidence intervals (CI). The heterogeneity among the included studies was examined with I^2^ statistics with I^2^ < 50% signifying homogeneity. Taking into account the heterogeneity in patient population and study design, all analyses were conducted based on the Mantel–Haenszel random-effects model. Meta-regression analysis was used to explore the source of heterogeneity as previously reported^29,30^. The likeliness of publication bias was assessed by funnel plots for outcomes reported in 10 or more studies. To examine the robustness of the results and identify the source of heterogeneity, sensitivity analysis was performed through a leave-one-out approach. The comprehensive Meta-Analysis (CMA) V3 software (Biostat, Englewood, NJ, USA) was adopted for all statistical analyses, in which significance referred to a probability value (*p*) less than 0.05.

## Results

### Study selection and quality

**Figure 1:**
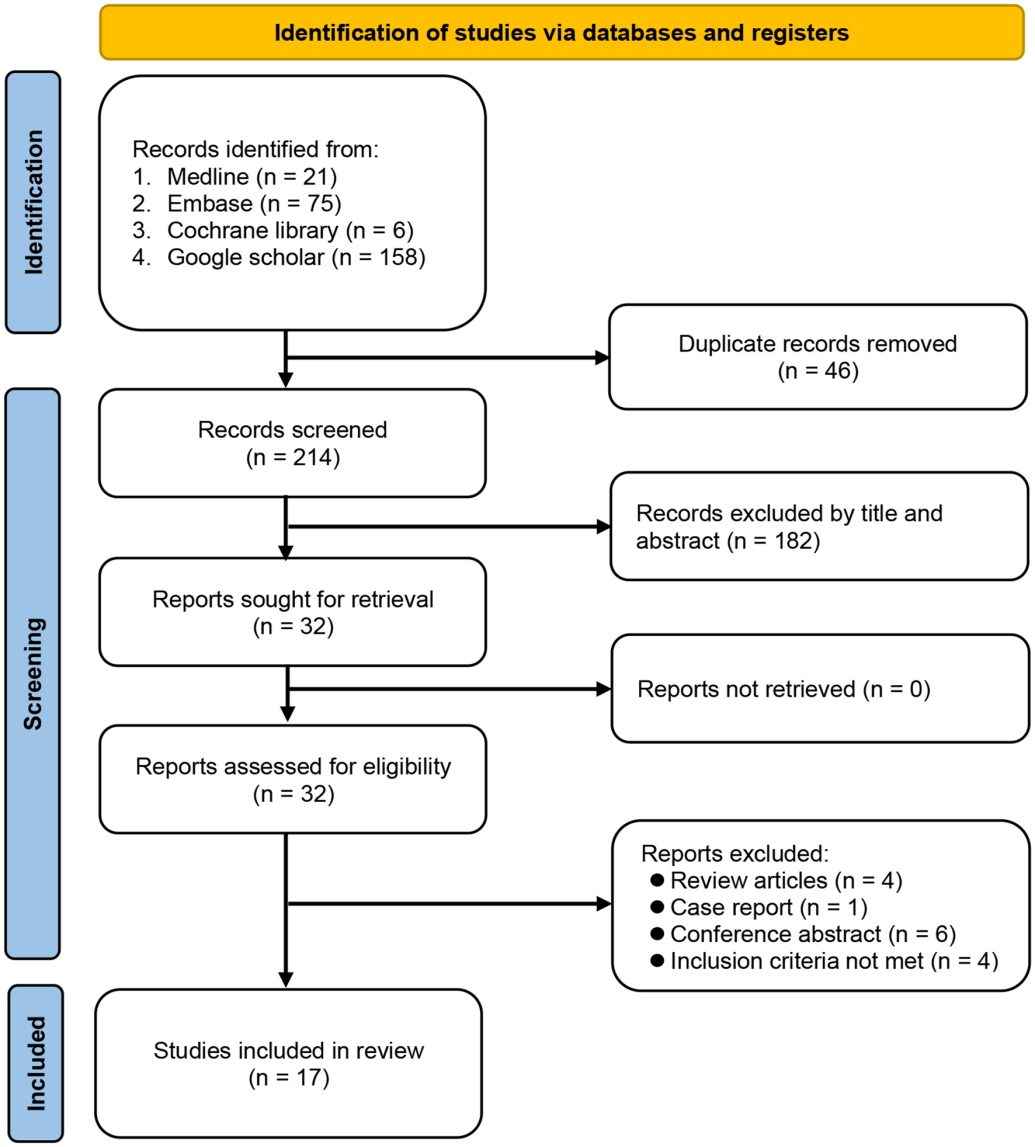
Selection process of studies based on search strategies.

Of the 260 records initially identified through a comprehensive search of the four different databases, 46 were duplicate publications and 182 did not meet the criteria for full-text reading. Reviewing the full text of the remaining 32 studies further excluded 15 publications based on a variety of reasons (Fig. 1). Finally, 17 articles published between 2015 and 2021 were deemed eligible for the current meta-analysis^14,15,26–28,31–42^. Figure 1 summarized the process to identify studies.

Characteristics of the 17 included studies with a total of 10,678 patients receiving TAVR are shown in Table 1. Of the 17 studies, 13 adopted a retrospective design^14,26,31–40,42^, three used a prospective design^15,27,41^, and one was a RCT^28^. The mean age of the patients ranged from 74.68 to 85 years, with a male gender distribution ranging from 36.9 to 65%. There was a wide variation in sample size across the included studies (range: 78 to 5248 patients). The information on body mass index (range: 23.18 to 28.4 kg/m^2^) was available in 12 studies, while it was unavailable in the other five studies^15,26,38,39,41^. Of 17 studies, 10 used validated methods for POD diagnosis^14,15,26–28,31,38–41^, namely the CAM, CAM-ICU, NU-DESC, DSM IV, and DSM V. Five studies did not report the diagnostic measures for POD^32–34,36,42^, and the two remaining studies based their diagnosis of POD on clinical symptoms (e.g., any acute disturbance of consciousness with decreased attention)^35^ or chart-based delirium identification instrument^37^, respectively.

**Table 1:** Characteristics of studies included (n = 17).

Study	Study design	Age (year)†	Male (%)	n	BMI	Diagnosis of POD	Overall incidence of POD (%)	EF%	Country	NOS
Abawi 2016	Retrospective	80	46	268	26	DSM IV	13.4	na	The Netherlands	7
Bagienski 2017	Retrospective	82	36.9	141	27.5	CHARTDEL	20.6	60	Poland	7
Gauthier 2015	Retrospective	85	48.7	117	25	Symptom^‡^	27.3	na	Belgium	8
Goudzwaard 2020	Prospective	79.1	55	543	27.3	DSM-IV	14.0	na	The Netherlands	7
He 2017	Retrospective	75.0	65	113	23.18	na	4.4	52.3	China	9
Husser 2018	Retrospective	81.6	43.6	5248	27	na	2.4	na	Germany	8
Kalyoncuoğlu 2020	Retrospective	76.3	41	78	na	CAM-ICU	21.8	47.8	Turkey	7
Khan 2019	Prospective	82.2	59.4	234	na	CAM-ICU	9.8	na	Canada	7
Lee 2021	Retrospective	79	49.6	589	23.9	CAM-ICU	6.6	na	Korea	8
Luque 2021	Retrospective	82.9	42.3	501	27.9	CAM	22.0	58 vs. 58	Spain	7
Maier 2020	Retrospective	81.2	46	308	na	NU-DESC	16.6	49 vs. 51	Switzerland	9
Mauri 2021	Prospective	82.3	48.7	661	na	CAM-ICU	10.0	na	Germany	8
Mosieh 2019	Retrospective	81	52.3	308	28.4	na	11.0	na	USA	9
Musuku 2021	Retrospective	81.3	59.5	256	27.5	na	0.8	na	USA	9
Renner 2019	Retrospective	82	46	200	26.2	na	4.0	na	Germany	9
Thiele 2020	RCT	81.6	48.9	438	26.8	CAM-IU	12.0	na	Germany	-
Wesselink 2021	Retrospective	81	46	675	na	DSM-V	14.0	na	The Netherlands	7

^‡^Any acute disturbance of consciousness with decreased attention; CHARTDEL: chart-based delirium identification instrument; CAM-ICU: Confusion Assessment Method for the Intensive Care Unit; CAM: Confusion Assessment Method; NU-DESC: Nursing Delirium Screening Scale; DSM IV: Diagnostic and Statistical Manual of Mental Disorders, 4th ed; DSM IV: DSM V: Diagnostic and Statistical Manual of Mental Disorders, 5th ed; na: not available; EF: ejection fraction; BMI: body mass index; RCT: randomized controlled trial.

The quality of cohort studies (n = 13) is summarized in Table 1. In brief, all 17 studies were considered to be of high quality (range of NOS: 7–9). For the only RCT^28^, the risk of bias is deemed low.

### Outcomes

#### Primary outcome: association of GA exposure with risk of POD

**Figure 2:**
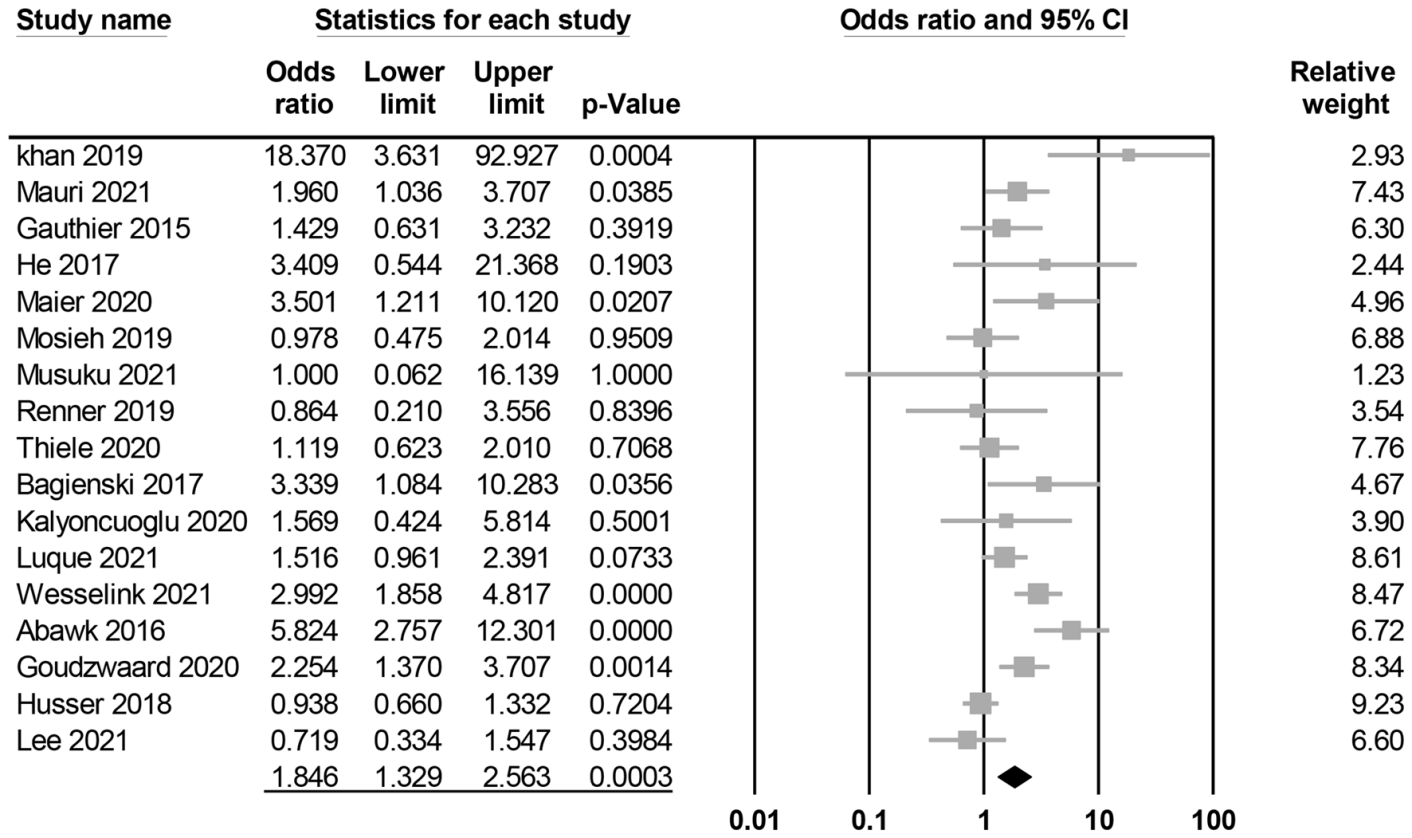
Forest plot showing the risk of postoperative delirium among patients with general anesthesia and those without.

**Figure 3:**
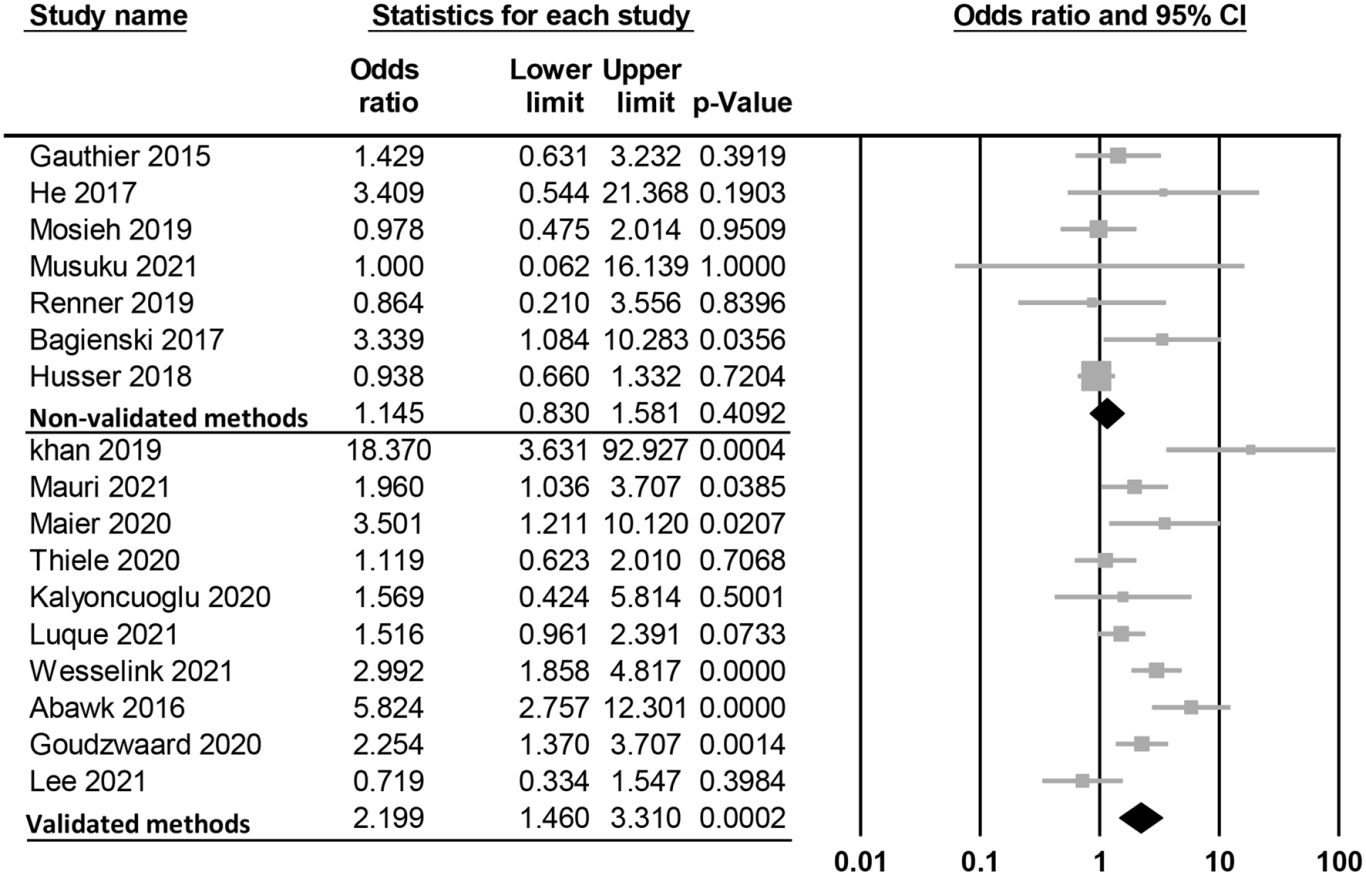
Subgroup analysis demonstrating the association of general anesthesia with the risk of postoperative delirium focusing on the use of validated versus non-validated diagnostic methods.

Meta-analysis of 17 studies revealed an association of GA with an elevated risk of POD (OR = 1.846, 95% CI 1.329 to 2.563, p = 0.0003, I^2^ = 68.4%, 10,678 patients) (Fig. 2)^14,15,26–28,31–42^. Sensitivity analysis confirmed the robustness of evidence (Supplemental Fig. 1). Subgroup analysis of the diagnostic methods also demonstrated a positive correlation between GA exposure and POD risk when the validated methods were used for POD diagnosis (OR = 2.199, 95% CI 1.46 to 3.31, *p* = 0.0002)^14,15,26–28,31,38–41^ (Fig. 3). However, there was no significant link between GA and the risk of POD when non-validated diagnostic methods were used (OR = 1.145, 95% CI 0.83 to 1.581, *p* = 0.4092) (Fig. 3)^32–37,42^. Publication bias was deemed low on funnel plot examination (Supplemental Fig.2).

**Figure 4:**
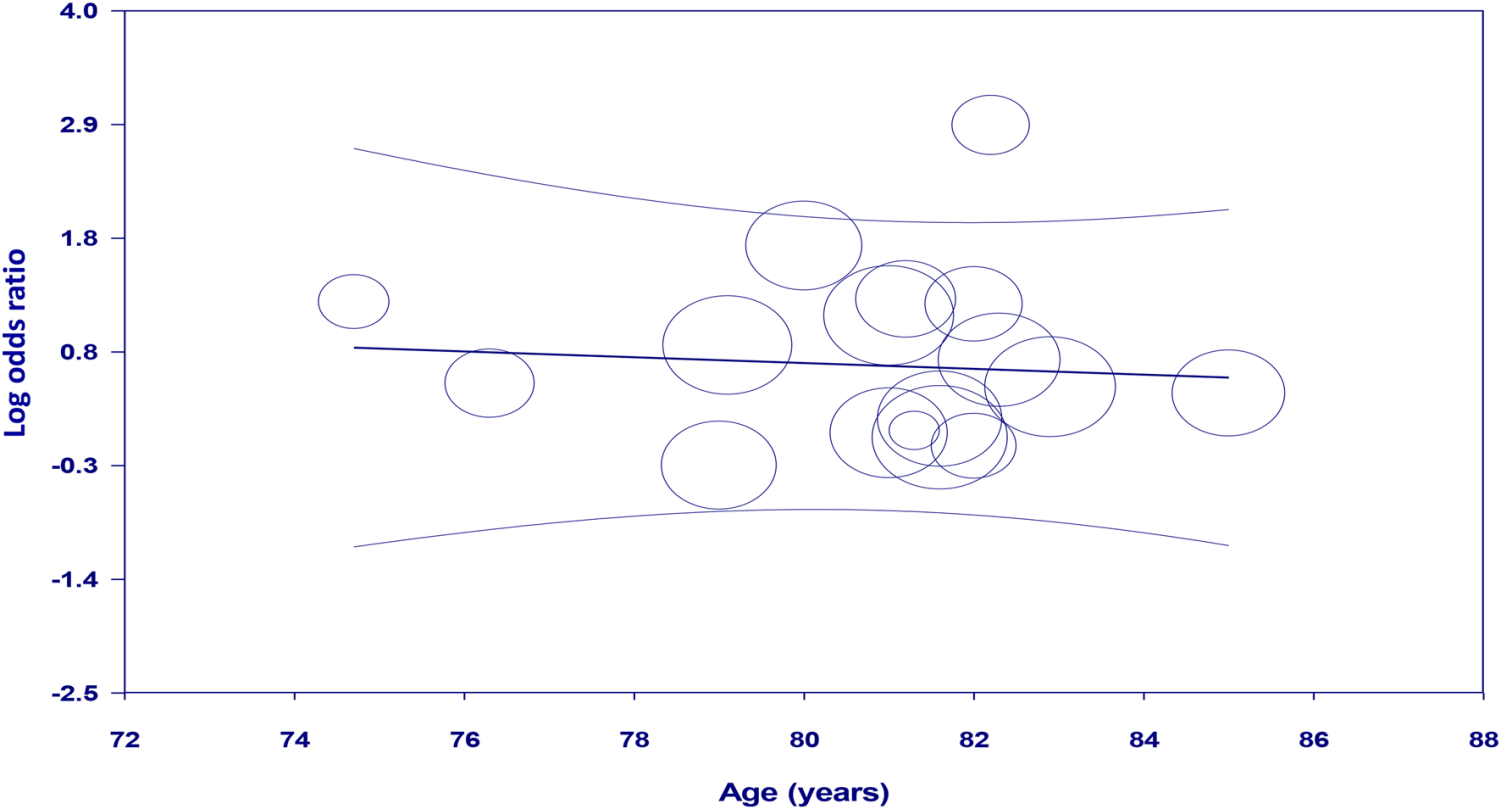
Meta-regression analysis showing non-significant correlation between patient age and study outcome.

**Figure 5:**
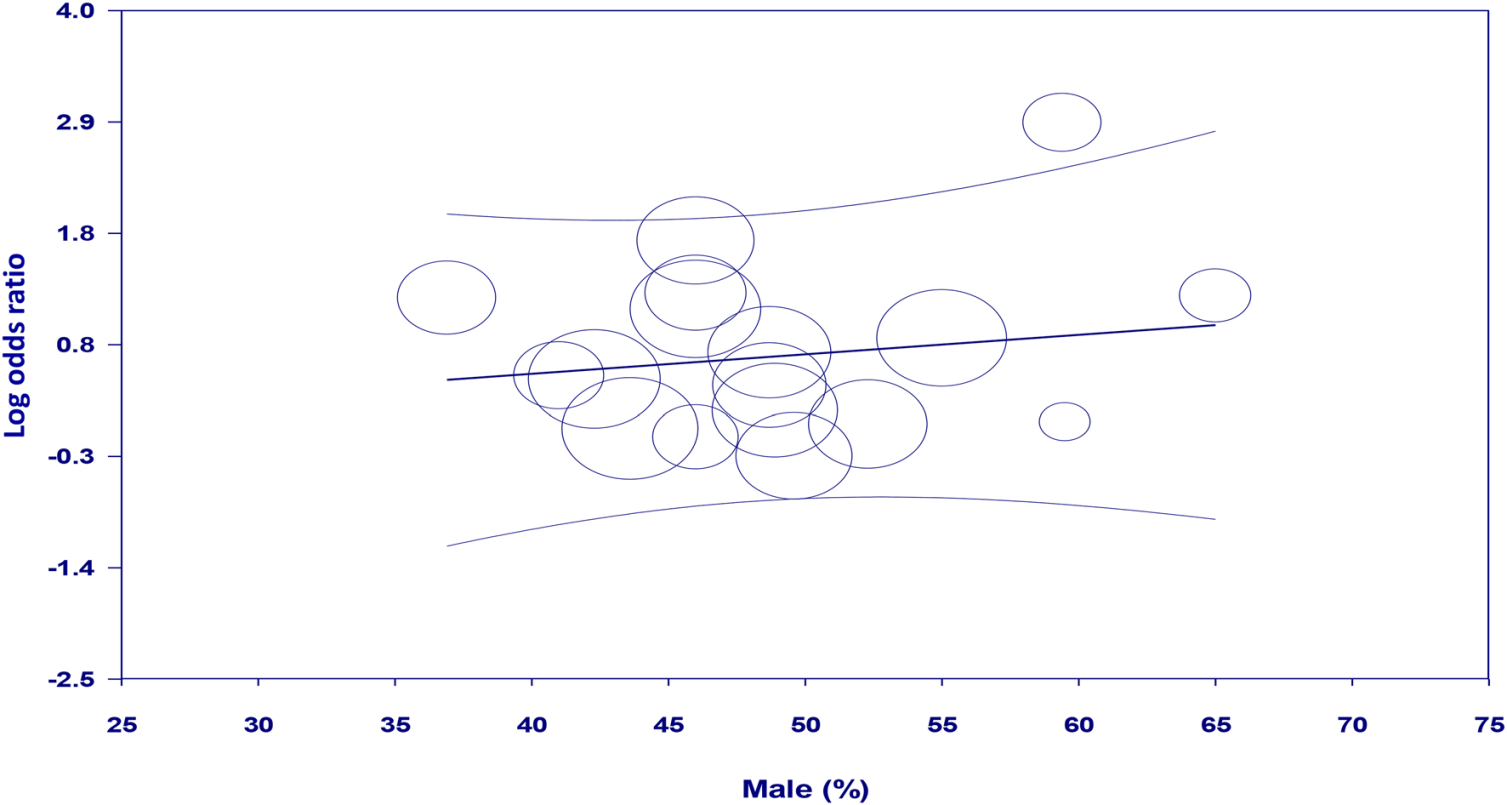
Meta-regression analysis indicating non-significant association between male proportion and study outcome.

**Figure 6:**
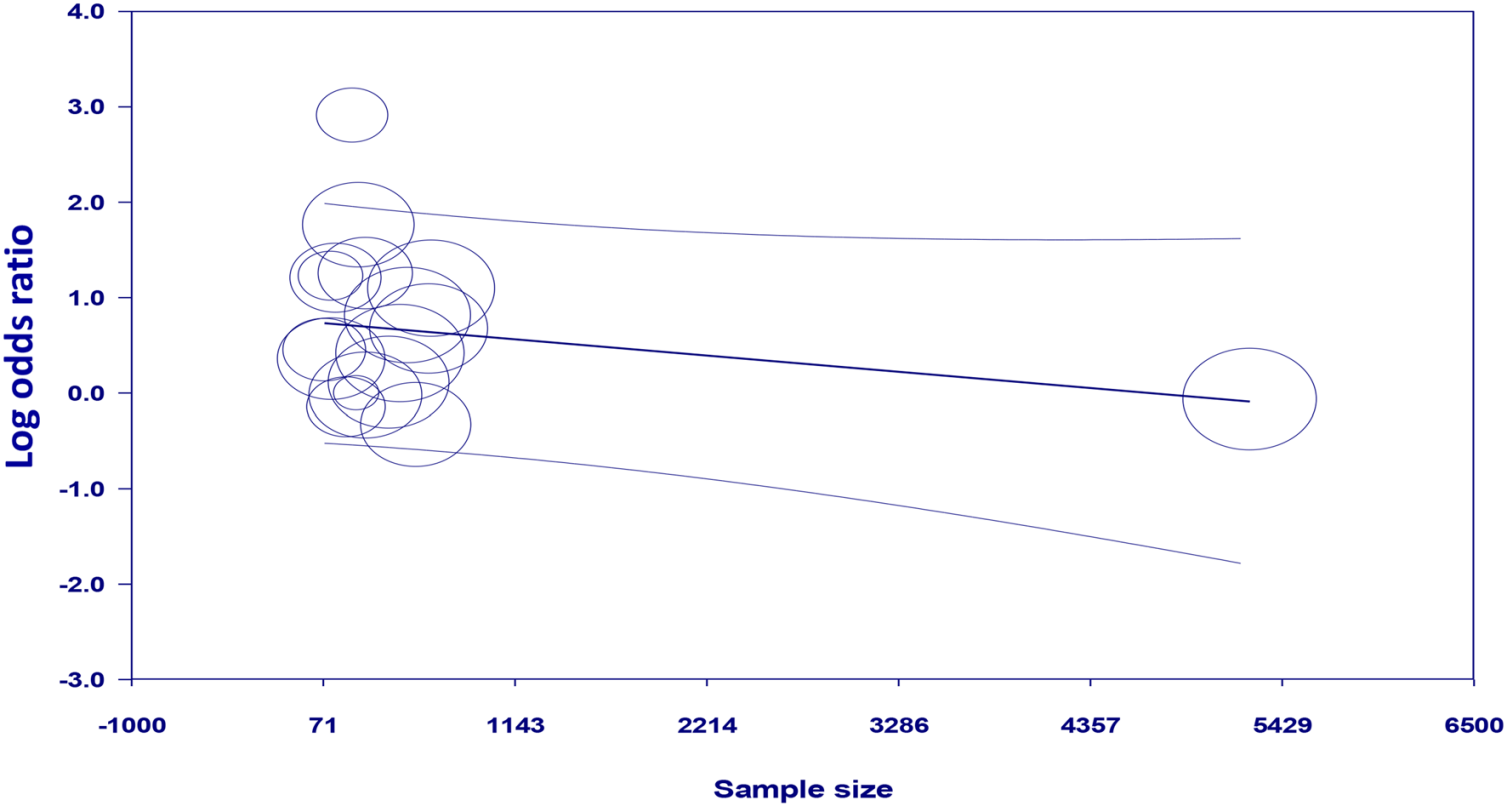
Meta-regression analysis demonstrating non-significant correlation between sample size and study outcome.

Meta-regression analyses showed no significant impact of age on the association between GA exposure and the risk of POD (coefficient: − 0.028, *p* = 0.742) (Fig. 4). There were also no significant influences of other covariates including male proportion (coefficient: 0.019, *p* = 0.53) (Fig. 5) and sample size (coefficient: -0.0002, *p* = 0.149) (Fig. 6) on the correlation between GA and POD risk.

#### Secondary outcome: incidence of postoperative delirium

**Figure 7:**
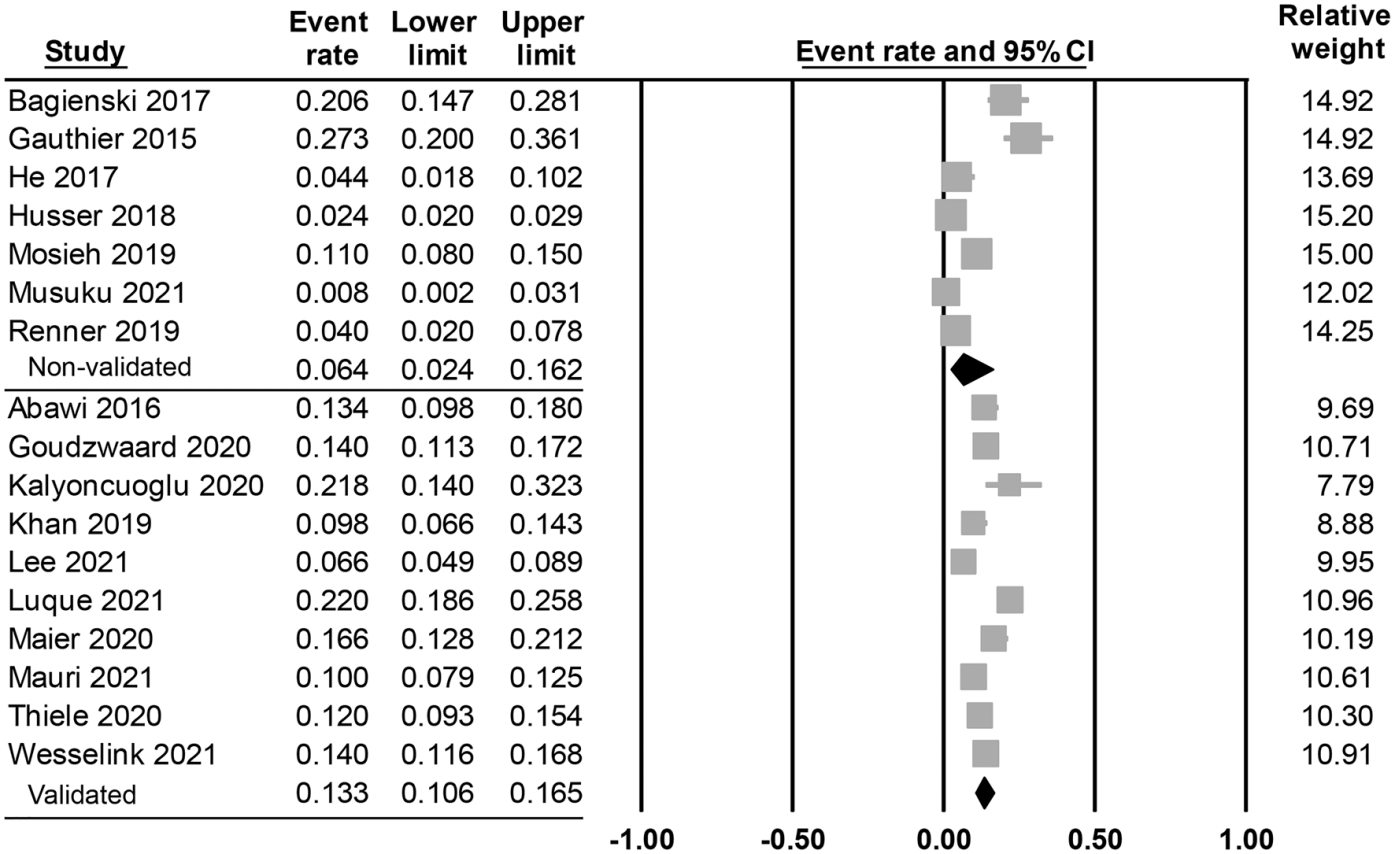
Forest plot showing the pooled incidence of postoperative delirium based on validated or non-validated methods for postoperative delirium (POD) diagnosis. CI: confidence interval.

The reported overall incidence of POD from the included studies regardless of the type of anesthesia was between 0.8 and 27% (Table 1). Our meta-analysis showed a pooled incidence of 10.3% (95% CI 7% to 15%) (Figure not shown). Subgroup analysis revealed a pooled incidence of 13.3% when focusing on validated methods for POD diagnosis, while it was 6.4% if non-validated methods were used (Fig. 7).

### Certainty of evidence

The certainty of evidence, which was downgraded due to a high heterogeneity (I^2^ = 68.4%), was considered very low for the primary outcome.

## Discussion

Through a meta-analysis of 17 studies involving 10,678 patients, the current study revealed a statistically significant association of GA exposure with an increased risk of POD in individuals undergoing TAVR. Sensitivity analysis demonstrated consistency of our result. Subgroup analysis based on studies that used validated tools for POD diagnosis also supported the finding. In addition, there was a low risk of publication bias in the current meta-analysis, indicating the robustness of our results. Furthermore, age, sample size, and male proportion had no significant influence on the correlation between GA exposure and POD risk according to meta-regression analysis. To our best knowledge, the present meta-analysis is the first extensive review to focus on the relationship between GA exposure and the risk of POD following TAVR.

Our analysis showed a pooled POD incidence of 10.3% after TAVR. The incidence of POD varies among different surgical populations. In patients receiving surgery for hip fracture, the incidence has been reported to be 16–62%^43^. For those receiving cardiac surgery, POD occurs in 8–31% of patients, ranging from 25 to 52% in those aged 60 or above and 31–66% in those aged over 70^14^. The etiology of POD comprises a complex combination of predisposing factors, including advanced age, pre-existing cognitive impairment, depression, vision impairment, previous stroke, and precipitating factors such as surgery, acute pain, malnutrition, and hospitalization^7^. The reduced incidence of POD in our patients undergoing TAVR could be attributed to the absence of factors associated with surgery or pain that might trigger it.

Besides inflammatory response that has been proposed to be a contributor to POD^7^, several modifiable and non-modifiable risk factors were identified in a recent large-scale meta-analysis of 69 studies focusing on patients undergoing TAVR without exposure to surgery-induced inflammatory response^11^. Non-modifiable predisposing factors included advanced age, prior stroke/transient ischemic attack, male gender, and postoperative acute kidney injury, atrial fibrillation/flutter, and impaired Instrumental Activities of Daily Living^11^, while modifiable risk factors consisted of GA exposure, weight loss, electrolyte imbalance, and techniques of TAVR (e.g., non-transfemoral Access)^11^. Despite the inclusion of up to 69 studies in that meta-analysis, it involved only nine studies with 3,555 patients when assessing the impact of GA on the risk POD^11^. In contrast, our investigation included 17 studies involving 10,678 patients. In addition, we conducted meta-regression analysis to explore the source of heterogeneity.

In our meta-regression analysis, the absence of age and sex influences on the GA-POD link is interesting. Advanced age and male sex are well-established risk factors for POD based on previous research^11^. The lack of association in this meta-regression analysis suggests that GA exposure may increase the risk of POD regardless of age and sex in patients undergoing TAVR. However, it should be noted that uncontrolled confounding factors (e.g., frailty) could have obscured any potential effects of age and gender on the GA-POD association. In addition, the lack of an association between age/sex and GA-POD risk may be due to insufficient heterogeneity in these covariates rather than a true lack of effect. More research is needed to control for relevant confounders to determine whether age or sex interacts with GA to modify POD risk after TAVR.

Although the correlation between GA exposure and an elevated risk of POD remains controversial^18–23^, it is generally believed that GA exposure would lead to an increased risk of POD; however, a large-scale RCT that randomly assigned patients to either GA or spinal anesthesia did not find any difference in the incidence of POD between the two groups^44^, suggesting no causality between GA exposure and the subsequent POD. On the other hand, several studies reported an association of a proinflammatory state with the development of POD^45–47^, highlighting inflammation as a potential underlying mechanism. Consistently, a previous meta-analysis of 54 observational studies revealed significantly increased circulating C-reactive protein and interleukin-6 concentrations in patients with POD^48^. Furthermore, intrinsic immune response activation has recently been proposed to be a mechanism underlying the occurrence of POD^49,50^. Such neuroinflammation, which can be triggered by surgical trauma or systemic diseases^51^, could result in neuronal dysfunction and death^52^.

There may be several possible explanations for the association between GA exposure and an elevated POD risk in the current meta-analysis (i.e., OR = 1.846). First, old age is a known risk factor for POD^11^. A previous study reported a positive correlation between an increasing age and the occurrence of POD with the incidence of POD being 22% in patients aged 50–59 but up to 92% in those aged 80–89^53^. Therefore, our inclusion of individuals undergoing TAVR, who are frequently elderly (i.e., age range: 74.68–85), may increase their susceptibility to POD. Second, compared to other anesthetic techniques (e.g., sedation or local anesthesia), the use of GA may lead to a higher risk of peri-procedural hypotension^54^, which is a potential risk factor for POD^55–57^. Third, a previous meta-analysis has reported that GA exposure may significantly increase the risk of prolonged mechanical ventilation and acute kidney injury^54^, which are also known risk factors for POD^58,59^. Fourth, besides advanced age, patients receiving TAVR are characterized by frailty, malnutrition, and extensive comorbidities^15^, which may render this population particularly vulnerable to the development of POD following GA exposure. In summary, the increased risk of POD in this patient population may be attributed to the interaction between GA exposure, advanced age, comorbidities, malnutrition as well as GA-related complications.

A significant association between GA and elevated risk of POD was observed in studies employing validated diagnostic tools, whereas studies utilizing non-validated methods did not show a significant correlation. We suggest that validated diagnostic tools specifically designed to identify delirium (e.g., CAM) may be more accurate and sensitive for detecting POD than non-standardized symptom-based evaluations. Therefore, studies using validated tools may identify a greater proportion of POD cases, strengthening the observed association between GA and POD risk. In contrast, non-validated chart-based screening methods are likely to have a lower sensitivity for POD diagnosis. Consequently, studies relying on these approaches may fail to identify all POD cases, underestimate the true incidence, and dilute the correlation between GA and risk of POD.

With accumulating evidence suggesting the feasibility of TAVR as a relatively low-risk therapeutic alternative to conventional surgery for patients diagnosed with severe symptomatic aortic valve stenosis^60–62^, TAVR is expected to gain increasing popularity among those who are at high risk for surgical repair. Although GA may be preferred over sedation/local anesthesia due to its ability to facilitate timely surgical correction and enable real-time monitoring through transesophageal echocardiography during the early learning curve development for TAVR, sedation/local anesthesia may be an optimal choice for patients receiving TAVR based on the finding of the current meta-analysis, especially for those at high risk of POD (e.g., anticipated prolonged mechanical ventilation). On the other hand, if GA is indicated (e.g., at the early stage of the learning curve), implementation of appropriate preventive strategies may be indicated. Non-pharmacological strategies including multicomponent interventions, avoidance of peri-operative benzodiazepines, minimizing precipitating events, using processed electroencephalogram monitoring (e.g., bispectral index) to guide anesthetics titration may be beneficial^63–65^, while strategies for pharmacological prophylaxis such as the use of antipsychotics and peri-procedural dexmedetomidine as well as optimizing postoperative pain control have also been reported^63,64,66^. Besides, a previous meta-analysis of six studies reported that perioperative melatonin, a natural hormone known to regulate sleep–wake cycles, could be effective for preventing POD in older patients^66^.

There were several limitations in the current study. First, because we mostly included retrospective studies, the causality relationship between GA exposure and POD risk could not be definitely established. Second, although confounding factors such as peri-procedural hypotension or history of cognitive impairment may contribute to this cognitive dysfunction, they were not investigated due to unavailable information. Third, the effects of other predisposing factors including advanced age, gender, and technique of TAVR, which have been well-described in a recent meta-analysis^11^, were not investigated in the present study. Third, our results, which were derived mostly from patients aged more than 80 years, may not be extrapolated to younger populations. Finally, we did not evaluate the long-term influence of POD as this information was not available in most studies.

## Conclusion

Our analysis of 10,678 patients over 75 years of age receiving TAVR showed an elevated risk of postoperative delirium following general anesthesia exposure with a low risk of publication bias and consistency of result on sensitivity analysis. The inclusion of mostly observational studies in the current study warrants further randomized controlled clinical investigations to support our findings and to elucidate the efficacy of possible preventive strategies (e.g., dexmedetomidine administration) against postoperative delirium in this clinical setting.

### Supplementary Information

Supplementary Information.

## Data Availability

The original contributions presented in this study are included in this article/Supplementary material, further inquiries can be directed to the corresponding author.
