# Supplementary material for: Association of general anesthesia exposure with risk of postoperative delirium in patients receiving transcatheter aortic valve replacement: a meta-analysis and systematic review
Source: Sci Rep. 2023 Sep 27;13:16241. doi: 10.1038/s41598-023-43548-2 (PMC10533830; doi:10.1038/s41598-023-43548-2)

**Supplemental Table 1.** Search strategies for Medline

| 1 | ("transcatheter aortic valve replacement" or "TAVR" or "Aortic valve stenosis" or "Transcatheter Aortic Valve Implantation" or "TAVI").mp. |
| --- | --- |
| 2 | exp "Transcatheter Aortic Valve Replacement"/ or exp "Aortic Valve Stenosis"/ |
| 3 | ("General anesthesia" or "Tracheal intubation*" or "Endotracheal Intubation" or "inhalation agents").mp. |
| 4 | exp "Anesthesia, General"/ or exp "Intubation, Intratracheal"/ or exp "Anesthetics, Inhalation"/ |
| 5 | ("delirium" or "postoperative delirium" or "Confusion Assessment Method" or "cognitive decline" or "cognitive dysfunction" or "cognition impairment" or "acute brain failure" or "acute brain dysfunction" or "altered mental status" or "organic brain syndrome").mp. |
| 6 | exp "Neurocognitive Disorders"/ or exp "Delirium"/ |
| 7 | (1 or 2) and (3 or 4) and (5 or 6) |

**Supplemental Figure 1.** Sensitivity analysis showing the robustness of evidence relating to the association between general anesthesia and postoperative delirium.


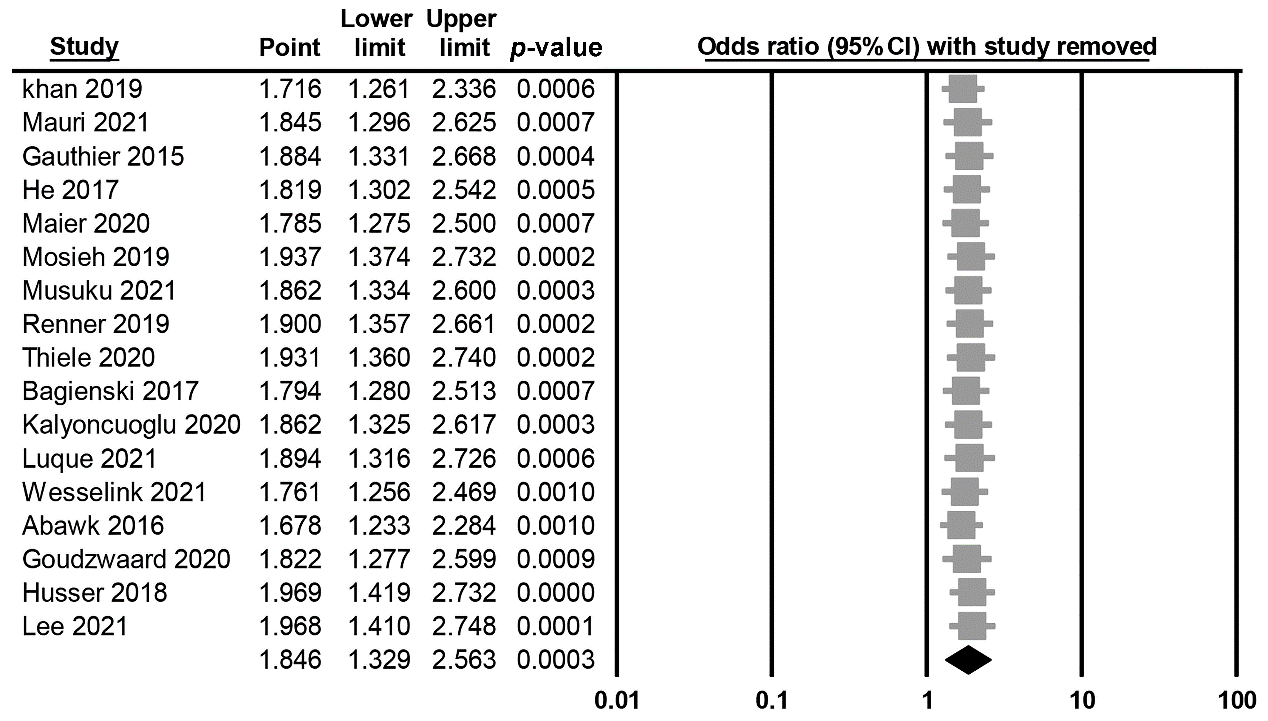


**Supplemental Figure 2.** Funnel plot indicating a low risk of publication bias.


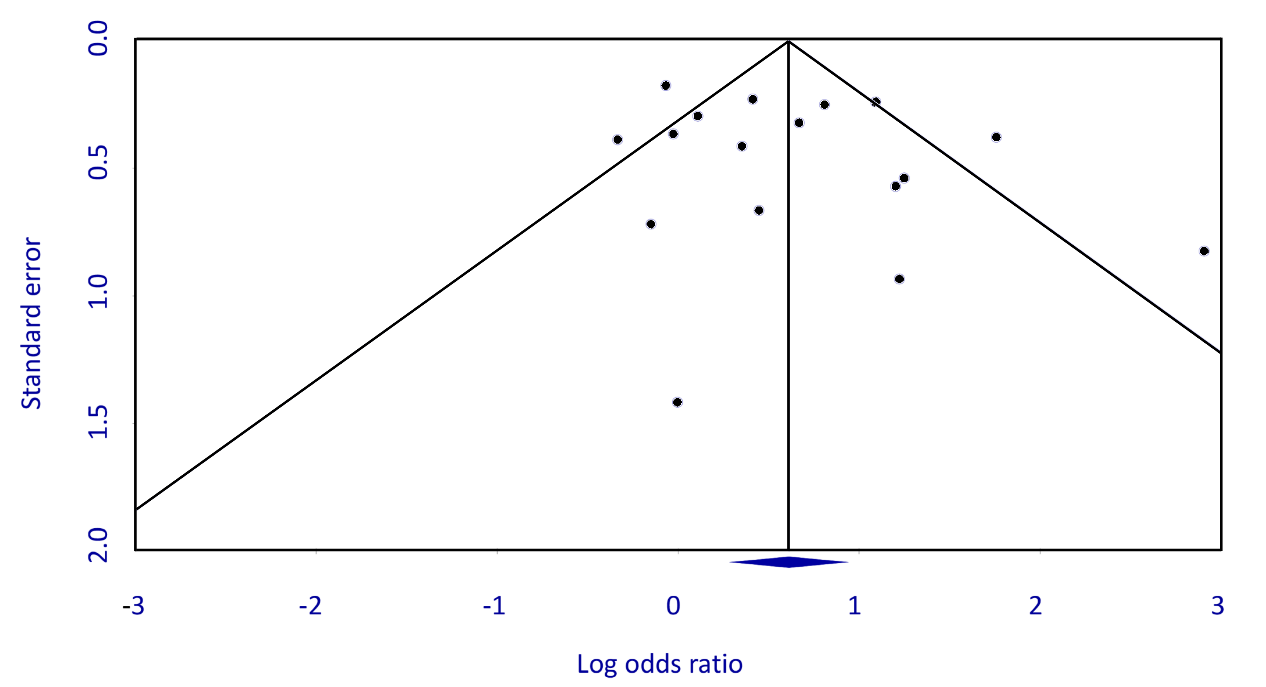

Supplement: Supplementary file 1 — Supplementary Information. [file 41598_2023_43548_MOESM1_ESM.docx]
